# Placebo response mitigation with a participant-focused psychoeducational procedure: a randomized, single-blind, all placebo study in major depressive and psychotic disorders

**DOI:** 10.1038/s41386-020-00911-5

**Published:** 2020-11-26

**Authors:** Elan A. Cohen, Howard H. Hassman, Larry Ereshefsky, David P. Walling, Vera M. Grindell, Richard S. E. Keefe, Katarzyna Wyka, William P. Horan

**Affiliations:** 1 Hassman Research Institute, Marlton, NJ USA; 2 Collaborative NeuroScience Research, Garden Grove, CA USA; 3 VeraSci, Durham, NC USA; 4 Duke University, Durham, NC USA; 5 The City University of New York, New York, NY USA; 6 University of California, Los Angeles, Los Angeles, CA USA

**Keywords:** Human behaviour, Drug discovery

## Abstract

The remarkably high and growing placebo response rates in clinical trials for CNS indications, such as depression and schizophrenia, constitute a major challenge for the drug development enterprise. Despite extensive literature on participant expectancies and other potent psychosocial factors that perpetuate placebo response, no empirically validated participant-focused strategies to mitigate this phenomenon have been available. This study evaluated the efficacy of the Placebo-Control Reminder Script (PCRS), a brief interactive procedure that educates participants about factors known to cause placebo response, which was administered prior to the primary outcome assessments to subjects with major depressive or psychotic disorders who had at least moderate depression. Participants were informed they would participate in a 2-week randomized clinical trial with a 50% chance of receiving either an experimental antidepressant medication or placebo. In actuality, all participants received placebo. Participants randomly assigned to receive the PCRS (*n* = 70) reported significantly smaller reductions (i.e., less placebo response) in depression than those who did not receive the PCRS (*n* = 67). The magnitude of this effect was medium (Cohen’s d = 0.40) and was not significantly impacted by diagnostic status. The number of adverse events (i.e., nocebo effect) was also lower in the PCRS group, particularly in the first week of the study. These findings suggest that briefly educating participants about placebo response factors can help mitigate the large placebo response rates that are increasingly seen in failed CNS drug development programs.

## Introduction

Drug development programs continue to face dauntingly low success rates in late phase randomized placebo-controlled trials (RCTs). Across all indications, the ultimate likelihood of approval for drugs entering Phase I is ~9.5%, with success rates of about 30.7% and 58.1% in late Phase II and III trials, respectively [1]. The situation is particularly dire for central nervous system (CNS) indications. For example, psychiatric medications are among the least successful, with a 6.3% ultimate likelihood of approval, success rates of 23.7% in Phase II and 55.7% in Phase III, and with substantially longer (about 2 years) Phase II and III development timelines than non-CNS indications [1, 2]. In most cases, these failures are due to a lack of efficacy in demonstrating that active treatment signal separates from placebo and is a crucial reason several pharmaceutical companies have reduced or even closed their psychiatric research and development programs [3–5].

Although placebo effects are a natural phenomenon and cannot be avoided completely in clinical trials, the placebo response is particularly large in major depressive disorder (MDD) and schizophrenia (SCZ). Placebo response rates of at least 30%, with variability ranging from 13 to 50%, are seen in RCTs in MDD with comparable rates of at least 25%, ranging from 6 to 41%, in SCZ [6–9]. Furthermore, placebo response rates have steadily increased over the past several decades [8, 10, 11], which has been described as, “a major obstacle in CNS drug development” [12], and a “growing crisis” [13]. At the same time, nocebo effects, which refer to undesirable effects (i.e., adverse events [AEs]) following administration of inert placebo treatment, are also substantially elevated in MDD and SCZ trials [14–16]. Nocebo effects substantially impact adherence and study withdrawal rates, further complicating efforts to statistically detect drug-placebo differences and bring potentially helpful compounds to market [17].

The large, variable, and increasing placebo response rates in late phase RCTs have led clinical trialists to investigate potentially modifiable causes of this phenomenon. Many contributing factors have been identified, which can be broadly categorized into participants’ internal psychological processes and external social contextual factors [18–20]. Chief among the psychological processes are participants’ treatment expectancies [6, 21–24], which refer to positive or negative expectations of the benefits of participation, as well as participants’ cognitive biases, understanding (or lack thereof) of placebo conditions, and personal histories (e.g., prior experience in RCTs). These psychological processes, whose neurobiological correlates have been extensively examined [18, 25], interact with a range of social contextual factors. These factors include elements of the social environment, such as raters’ interpersonal qualities (e.g., warmth, authority) and communication styles (e.g., how measured they are in describing potential treatment benefits), as well as the treatment itself (e.g., placebo appearance and mode of administration), and the treatment setting (e.g., formality of the research site environment and clinicians).

Despite the extensive literature on participant-related factors that amplify placebo responses, to the best of our knowledge, there have been no efforts to develop and rigorously evaluate participant-focused strategies that directly address these factors in a clinical trial context. To date, placebo mitigation efforts have focused on clinical design features, such as Sequential-Parallel Comparison Design, using centralized/remote raters, as well as incorporating lead-in procedures, and their success in psychiatric populations has been decidedly mixed [26–28]. Grounded in the extensive research on psychosocial determinants of placebo response, the Placebo-Control Reminder Script (PCRS) was designed to systematically address key participant-related factors associated with enhanced placebo response. A trained rater reads a brief passage (script) to the participant that provides psychoeducation about (a) a set of factors shown to amplify placebo responses, including expectation biases related to treatment benefit, misunderstanding the purpose of a placebo controlled trial, and misunderstanding how interactions with clinical trial staff differ from those with mental health treatment providers, and (b) the importance of attending to, and guarding against, these potential biases and misconceptions when reporting on their symptoms and experiences. The rater then collaboratively queries the participant about his/her comprehension and responds to any misunderstandings. The PCRS is administered prior to the primary outcome measure at the beginning of a trial and then at each subsequent key study visit, which encourages sustained attention to, and defense against, placebo response related factors. The goal of the PCRS is to enable participants to serve as more balanced, impartial, and well-informed reporters of their symptoms and experiences during the course of a trial. This approach is intended to minimize some key sources of noise that can inflate placebo/nocebo responses, and thereby impede treatment signal detection.

This study evaluated the efficacy of the PCRS in a cohort of patients with MDD or a psychotic disorder who had at least moderate levels of depression. It was hypothesized that participants who received the PCRS would show a smaller decrease in depression than those who did not receive the PCRS. We also examined whether the groups differed in terms of nocebo responses and subjective beliefs about their response to the study treatment.

## Methods

### Participants

Participants were recruited from two clinical trial sites on the east and west coasts of the United States (US). Participants had a primary Diagnostic and Statistical Manual of Mental Disorders-Fifth Edition (DSM-5) diagnosis of either MDD or SCZ/schizoaffective disorder (collectively referred to as “SCZ”) based on each site’s standard study psychiatric screening interview conducted by experienced clinical research raters, as well as a comprehensive review of all available medical records. The study protocol implemented inclusion/exclusion criteria that mirror RCTs for these indications. Key inclusion included (but were not limited to): men and women between 18–65 years old; in a current major depressive episode according to DSM-5 with at least moderate depression (≥20) on the Beck Depression Inventory-II (BDI-II; [29]); BDI-II Item #9 (suicidal thoughts or wishes) score = 0; outpatient with no hospitalization for worsening of any mental health symptoms within six months; able to provide informed consent. SCZ participants were also required to be on at least one antipsychotic medication at the same dose for ≥30 days. For all subjects, key exclusion criteria included: initiated, terminated, or had a dose change of any psychiatric medication in the past 30 days; initiated, terminated, or changed any psychosocial intervention or planned to make any such changes within 6 weeks of the Screening Visit; met DSM-5 criteria for such disorders as bipolar disorder, schizophreniform disorder, persistent depressive disorder, primary substance-induced psychotic disorder, any personality disorder, and dementia; had moderate or severe alcohol and substance use disorder per the DSM-5 within 6 months.

### Procedures

This 2-week randomized, single-blind, all placebo study was an independent prospective study. Participants were informed via an IRB-approved Informed Consent Form (ICF) that they would be in a double-blind RCT of an experimental antidepressant medication. They were informed that they had a 50% chance of being randomized to either active medication or placebo. However, as part of the methodology, all participants received placebo. Since deception was necessary to assess placebo and nocebo effects in relation to the PCRS, the study procedures incorporated a number of IRB-approved ethical safeguards. Trained and experienced investigators carefully evaluated whether each participant was capable of providing informed consent via standard operating procedures implemented at the research sites, and the ICF stated that the study entailed some deception to obtain valid results, and as such, the full purpose of the study could not be revealed at the time the subject was participating in the trial. Participants were carefully monitored for safety and AEs throughout the study. At the end of the study, participants were fully debriefed via a formal IRB-approved Debriefing Form which clearly explained the purpose of the PCRS and that all participants received the placebo and not the active medication. Participants were encouraged to ask any questions about this aspect of the study and were provided contact information to follow up with any later questions or concerns. The subjects signed the Debriefing Form indicating they understood its content and the form was also signed by the delegated investigator. A copy of the form was provided to the subject. The copy of the form was mailed to early withdrawal participants who did not show for the last visit so they too were fully aware of the study’s purpose, design, and deception. All subjects were fully evaluated for safety by designated qualified clinicians at the conclusion of their participation, and appropriate referrals or resources for follow-up treatment were provided.

Unbeknownst to participants, they were randomized to either a PCRS group or a Non-intervention Group (NG) that never received the PCRS. The PCRS group received the PCRS procedure prior to completing the primary outcome measure, the BDI-II, at each study visit. Randomization was stratified by diagnostic status, age, and sex. As is typical in RCTs, best efforts were made to ensure the same rater administered the PCRS and the BDI-II at each visit. Aside from the PCRS, the study procedures were identical for both groups.

There were three study visits. Visit 1 included screening, collecting demographic and clinical history data, assessing depression on the BDI-II, and administering the Investigational Product (IP). Visit 2 (end of week 1) included assessing depression via the BDI-II, AEs, subjective beliefs, and the second administration of the IP. Visit 3 (end of week 2) involved assessing depression on the BDI-II, AEs, subjective beliefs, as well as providing the formal debriefing. Regarding the IP, delegated staff (e.g., the investigators or study coordinators) administered and watched participants ingest two one-inch white blinded (placebo) capsules once at Visit 1 (once all entry criteria were met) and again at Visit 2 at the site. We opted to administer the IP at the site rather than daily at home to avoid potential confounds associated with the notable non-adherence rates with home administration that many clinical trials experience [30].

### Placebo-control reminder script (PCRS)

The PCRS was designed to educate clinical trial participants about commonly cited factors that impact placebo responses and the importance of attending to these factors when reporting on symptoms and potential side effects. In this study, a trained rater read the PCRS, which was a scripted brief paragraph, to the participant that carefully and explicitly reviews the following placebo response factors: (a) the double-blinded nature of a placebo-control study, which means that site staff have no expectations about their improvement (e.g., “*We have no expectations of how you should or should not be feeling”*); (b) that no staff will be disappointed if they report symptomatically feeling better, worse, or the same; (c) that they as research participants should similarly have no expectation of improvement or worsening since it is unsure if the study compound is even effective (e.g*., “Also keep in mind that because this is a research study, it is unknown if the active study medication is actually effective”*); (d) subjects should feel no pressure to report certain symptoms and that their role as research partners is simply to honestly describe their experiences; (e) what a placebo pill is and what that means in regard to their role in the study (e.g., “*Remember that the placebo is inactive and should do nothing to help your symptoms and should cause no side effects”)*; and (f) how site staff will interact with them differently than their mental health clinicians or primary physician (i.e., a neutrality approach). The participant is then asked to describe his/her understanding of the PCRS content (“*And just to make sure we are on the same page, can you please tell me in your own words what I just told you?”*). The rater collaboratively uses standard probes as needed to respond to any participant misunderstandings and help ensure participant comprehension. The goal is to enable participants to become impartial research partners with the investigators in a joint effort to identify potentially beneficial new treatments. The entire procedure takes about 3 minutes.

### Assessments

#### Depression

Given the single-blind study design (staff were aware of the study’s purpose and methodology), we selected a well-established patient reported outcome measure as the primary endpoint. The BDI-II [29] has the combinatory characteristics of stability and malleability needed to evaluate the placebo response. Depression was also the dependent variable for the SCZ subjects because, while positive and negative symptoms are more common endpoints in trials for SCZ, self-report measures for these symptoms are not well-established or commonly used. Further, depression is highly prevalent (40–60%), associated with an array of poor clinical outcomes, and a common treatment target throughout the course of SCZ [31, 32]. The BDI has been extensively used in both MDD and SCZ and shows strong correlations with standard clinician reported depression outcome measures [33, 34].

### Adverse events

AEs (i.e., the nocebo effect) were systematically evaluated by trained raters using standard clinical trial procedures. AEs that were spontaneously reported, elicited, or observed were recorded with the start date, stop date, if the AE is ongoing, the severity of the AE, actions taken as a result of the event, outcome of the event, and whether the AE qualifies as serious AE. MedDRA^®^ was used as the standard coding dictionary for AEs.

### Subjective beliefs

Participants rated the overall degree to which their sadness/depression had improved or not improved since the beginning of the study on a 5-point scale. Responses were trichotomized during data analysis as worsened, stayed the same, or improved. Based on the blinding survey by Bang et al. [35], participants were also asked their opinion about which treatment, active medication or placebo, they received on a 4-point scale. Responses were dichotomized during analysis as active medication or placebo.

### Statistical analyses

Change in BDI-II scores across Visits 1–3 was analyzed using repeated-measures analysis of co-variance (RM-ANCOVA) that included group as the between-subjects factor, visit as a within-subject factor, and baseline BDI scores as a covariate. As attrition was low (7%), we opted not to conduct imputations for missing data. Effect sizes were computed by subtracting the mean change score (Visit 1–Visit 3) in the PCRS from the NG, and dividing by the pooled standard deviation of change scores. We also examined whether responder rates on the BDI-II (i.e., minimal clinically important difference: ≥ 17.5% [36]) differed between groups with a chi-square test. Finally, to examine whether the results were impacted by diagnosis, a RM-ANCOVA examined the three-way interaction of group-by-visit-by-diagnosis. Chi-square tests evaluated group differences in AEs at Visit 2 (since Visit 1) and Visit 3 (since Visit 2), and subjective beliefs reported at Visit 3.

## Results

### Demographics and baseline characteristics

**Fig. 1:**
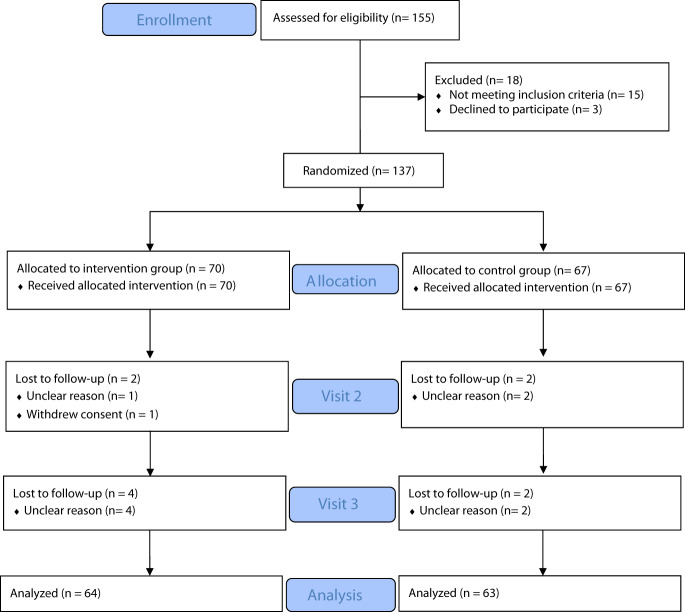
CONSORT flow diagram.

As shown in Fig. 1, a total of 137/155 screened patients were randomized to the PCRS (*n* = 70) or the NG (*n* = 67). Ten patients (7%) discontinued the study, including 6 (9%) in the IG and 4 (6%) in the NG. There were no significant group differences on demographics, baseline BDI-II scores, diagnosis, or clinical characteristics (Table 1).

**Table 1:** Baseline demographic data, clinical characteristics, and BDI-II scores.

	PCRS group (*n* = 70)	Non-intervention group (*n* = 67)	Statistic
Age (years)	46.43 (SD = 12.53)	46.48 (SD = 13.58)	*t*(135) = 0.02, *p* = 0.98
Sex (% male)	55.7%	49.3%	*χ*^2^ (1, *N* = 137) = 0.57, *p* = 0.45
Race			*χ*^2^ (2, *N* = 137) = 0.91, *p* = 0.64
White	24.3%	31.3%	
African American	62.9%	58.2%	
Other	11.7%	10.4%	
Education			*χ*^2^ (1, *N* = 137) = 0.25, *p* = 0.62
≤secondary education	80%	83.%	
>secondary education	20%	16.7%	
Site			*χ*^2^ (1, *N* = 137) = 0.14, *p* = 0.71
East cost	37.7%	38.8%	
West coast	64.3%	61.2%	
Diagnosis			*χ*^2^ (1, *N* = 137) = 0.02, *p* = 0.89
Major Depressive Disorder	61.4%	62.7%	
Schizophrenia	38.6%	37.3%	
Currently on psychiatric medication	68.6%	64.2%	*χ*^2^ (1, *N* = 137) = 0.30, *p* = 0.59
Previous trial participation	42.9%	49.3%	*χ*^2^ (1, *N* = 137) = 0.56, *p* = 0.45
Currently in psychotherapy	27.1%	19.4%	*χ*^2^ (1, *N* = 137) = 1.15, *p* = 0.28
Body Mass Index	31.51 (SD = 7.23)	31.88 (SD = 8.19)	*t*(135) = 0.28, *p* = 0.77
Baseline BDI-II	30.24 (SD = 8.52)	28.60 (SD = 6.84)	*t*(135) = −1.24, *p* = 0.22

*BDI-II* Beck Depression Inventory-II, *PCRS* Placebo Control Reminder Scale.

### Impact on BDI scores

**Fig. 2:**
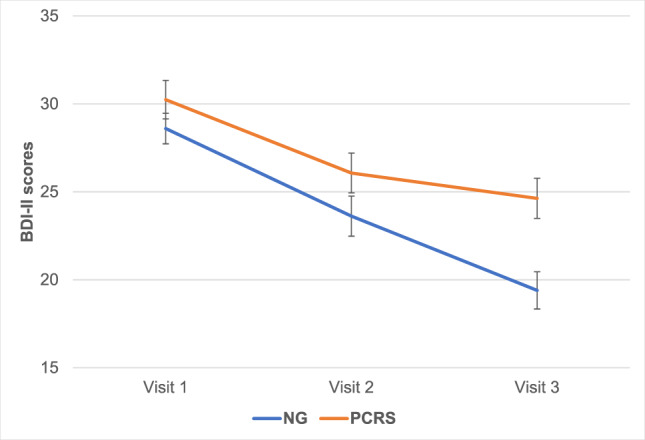
Mean BDI-II scores across visits. The overall linear decrease was significantly smaller in the PCRS group than the NG. BDI Beck Depression Inventory, PCRS Placebo Control Reminder Script, NG Non-intervention Group. Note: Error bars reflect standard errors.

As displayed in Fig. 2, BDI-II scores showed a significant overall linear decrease across visits, *F*(1,124) = 7.86, *p* = 0.006. The degree of change significantly differed between groups, *F*(1,124) = 9.81, *p* = 0.002, with a smaller decrease in the PCRS than the NG. The effect size of the group difference from Visits 1 to 3 was medium (*d* = 0.40). In line with this result, the proportion of patients that showed a minimal clinically important improvement on the BDI-II from Visit 1 to 3 was also significantly smaller in the PCRS (37.3%) than the NG (69.2%), *χ*^2^ (1, *N* = 137) = 12.50, *p* < 0.001. Diagnostic status did not have a significant impact on the overall pattern of BDI-II results, *F*(1,122) = 0.10, *p* = 0.75), though the effect size of the PCRS vs. NG difference from Visits 1 to 3 was larger within the SCZ subsample (*d* = 0.85; group X visit interaction: *F*[1,43] = 10.61, *p* = 0.002) than within the MDD subsample (*d* = 0.28; *F*[1,78] = 3.96, *p* = 0.05). As a cross-check, we also analyzed the BDI-II data with a mixed-effects model for repeated measures (MMRM) using all available data from all randomized subjects (modified intent-to-treat sample; *N* = 137) (see Supplementary methods and Supplementary Fig. 1). The results similarly indicated that the degree of change on the BDI-II from Visit 1 to Visit 3 significantly differed between the PCRS vs. NG (visit-by-group interaction, *t*[257] = 2.18, *p* = 0.03, and that diagnostic status did not impact the overall pattern of results (condition-by-visit-by-diagnosis interaction: *t*[258] = 1.00, *p* = 0.30) (Supplementary Table 1).

### Adverse events

**Fig. 3:**
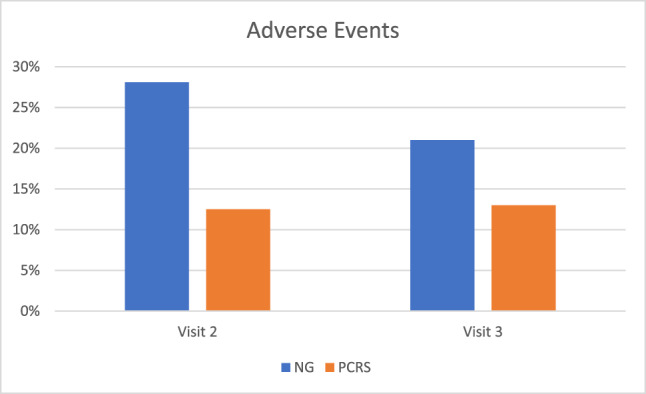
Adverse events reported at Visit 2 and at Visit 3. The percentage of reported adverse events was significantly lower in the PCRS group than the NG at Visit 2. The percentage of reported adverse events did not significantly differ between groups at Visit 3. PCRS Placebo Control Reminder Script, NG Non-intervention Group.

As shown Fig. 3, the proportion of patients reporting AEs at Visit 2 was significantly smaller in the PCRS (12.5%) than the NG (28.1%), *χ*^2^ (1, *N* = 128) = 4.82, *p* = 0.03. At Visit 3, the proportion that reported AEs was again numerically smaller in the PCRS (12.5%) than the NG (20.6%), but was not significant, *χ*^2^ (1, *N* = 127) = 1.52, *p* = 0.22.

### Subjective beliefs

**Fig. 4:**
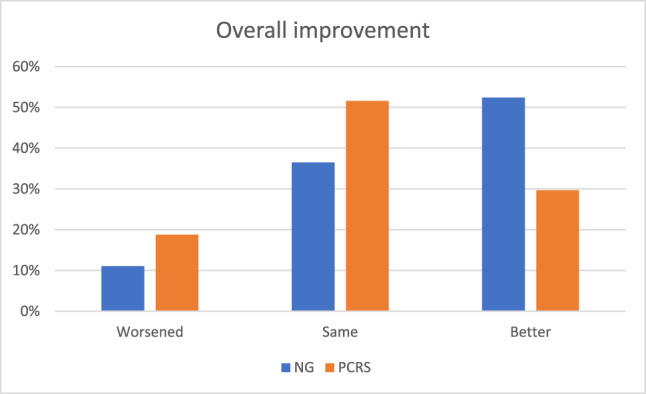
Subjective beliefs about performance. Compared to the NG, a significantly larger proportion of the PCRS group reported staying the same and a significantly smaller proportion of the PCRS group reported getting better. PCRS Placebo Control Reminder Script, NG Non-intervention Group.

At Visit 3, a significantly smaller proportion subjectively reported an overall improvement in depressive symptoms in the PCRS (29.7%) than the NG (52.4%), *χ*^2^ (1, *N* = 127) = 6.83, *p* = 0.03 (Fig. 4). The groups did not differ in the proportion of patients who believed they were in the active medication (PCRS: 56%, NG: 66%) vs. placebo (PCRS: 34%, NG: 44%) conditions, *χ*^2^ (1, *N* = 137) = 1.35, *p* = 0.25 (A supplemental analysis indicated that the self-reported active medication group reported significantly larger BDI-II score decreases (*F* = 25.35, *p* < 0.001) and a higher number of AEs (*F* = 21.93, *p* < 0.001) than the self-reported placebo group).

## Discussion

Depressed individuals with MDD or SCZ who received the PCRS reported significantly smaller reductions in depressive symptoms after receiving an inert substance than those who did not receive the PCRS. That is, the PCRS group responded less to receiving an inert substance (i.e., a reduced placebo response) and continued to report more depressive symptoms. Consistent with this finding, a smaller proportion of patients in the PCRS group also subjectively reported an overall improvement in symptoms. Thus, by initially educating and subsequently reminding participants about key factors known to amplify placebo response, we found a systematic reduction in symptom reports and global subjective impressions of change over the study period.

Failure to demonstrate that an active treatment separates from placebo is the most common cause of negative Phase II and III trials [1, 2]. The medium effect size of the PCRS vs. NG comparison, which corresponded to a between-group difference of ~5 points on the BDI-II, could substantially impact the outcome of a late phase trial. A number of pivotal trials have failed to achieve statistically significant separation from placebo by slim margins that would fall within the range (*d* = 0.40) of reduced placebo response associated with the PCRS [12, 37].

A variety of complicated and expensive clinical trial design features, such as double-blind placebo run-in periods and remote raters, have thus far failed to show clear and consistent benefits for mitigating the placebo response [22, 38]. Although trials sometimes provide placebo response educational slide shows or videos to raters, and to a lesser extent, to study participants, when launching a trial, there are usually no follow-up activities to help ensure that placebo mitigation strategies are continuously implemented. Further, as far as we are aware, there has been no evaluation of whether such strategies actually reduce placebo response using controlled, randomized procedures in a psychiatric sample with depression. The PCRS is a simple approach that involves providing education not only at the beginning of a trial but repeatedly throughout a trial. Notably, although the script is directed at the participant, it concurrently serves as a reminder to raters to manage their own behaviors and expectations—factors which have also been shown to enhance placebo response. Thus, the PCRS is an easy to administer and implement systemic tool which helps ensure participants’ as well as raters’ sustained attention to the factors known to perpetuate this phenomenon.

There was also some evidence that the PCRS impacted nocebo responses. The PCRS group reported lower levels of AEs than the NG, particularly at Visit 2. This suggests the impact of the PCRS may extend beyond participants’ evaluation and report of treatment benefits, and also impact how they perceive and report on undesirable experiences. Practically speaking, lower levels of AEs can translate into higher retention and adherence rates, and further enhance power to detect an active treatment signal [17].

Regarding subjective beliefs about treatment group assignment, it was somewhat surprising that the groups did not significantly differ in the proportions that believed they were randomized to active treatment. Although the proportion was somewhat lower in the PCRS (56%) vs. NG (66%), one might have expected a more pronounced difference in light of the substantial impact of the PCRS on the BDI-II and subjective reports of improvement. Making a categorical overall judgement about whether or not one received active medication may be a somewhat more abstract, complex process than reporting on one’s thoughts, feelings, and behaviors associated with assessing depression during the past week on a measure like the BDI-II. Perhaps these differences contributed to the lack of tight coupling between participants’ evaluations of treatment condition and reported improvements on the BDI-II.

Diagnostic group status did not significantly affect the overall results, indicating a beneficial PCRS effect across both MDD and SCZ. A supplemental analysis indicated that the impact of the PCRS was larger in the SCZ than the MDD group. It is noteworthy that this difference largely reflected variability on BDI-II change scores that was approximately twice as large in the MDD than in the SCZ subsample. The reason for this greater variability is unclear but could reflect factors such as the relatively higher baseline BDI-II scores in the MDD sample or the well-known heterogeneity of MDD [39, 40]. Alternatively, this difference could suggest that some of the placebo response factors addressed by the PCRS are more important for SCZ than MDD; further investigation of common and potentially unique placebo response factors between these disorders is needed. In any case, the PCRS impact on placebo response within SCZ and MDD was large enough to have shifted a number of prior negative trials in a positive direction.

One could question whether approaches like the PCRS might actually serve to diminish the ability to detect drug response vs. placebo. For example, factors such as expectancies and hope for treatment benefit may be an inherent component of a true treatment response to an active compound (e.g., [41]). Our results show that the PCRS did not fully eliminate a placebo response; participants in the PCRS group reported a significant reduction (albeit smaller than the non-PCRS group) in depression despite the absence of any active treatment. The PCRS is intended to systematically draw participants’ attention to potential sources of bias and misconceptions, enabling them to be more balanced, impartial reporters of their symptoms. Currently, these participant-level factors are simply not, or only superficially, addressed in clinical trials and essentially left to chance. We believe that systematically addressing this very important issue is more like to decrease noise than to decrease treatment signal detection, but we acknowledge this is an open question for this new area.

Although this study provides a rigorous evaluation of the PCRS, some limitations should be considered. First, the IP was administered once a week over a 2-week period with three assessment points, whereas CNS trials often involve daily IP administration over longer periods with more assessment visits. Second, the study focused on self-reported depression and did not evaluate whether benefits extend to clinician rated outcomes. In light of evidence that the placebo response is about three times larger for clinician- vs. self-ratings of depression [8], we expect it would. Third, depression is a less common trial focus in SCZ than positive and negative symptoms. Fourth, the study was only conducted within the US, which tends to show particularly large placebo responses [10]; the impact of culture on placebo response has received limited attention [42, 43] but is important in the context of increasingly global trials. Finally, it should be noted that the placebo response problem is only one of many complex methodological challenges that adversely impact signal detection in contemporary CNS trials [37, 44, 45].

In summary, the PCRS appears to be a useful tool to help mitigate the placebo response, which persists in plaguing late phase clinical trials for CNS disorders. Aside from MDD and SCZ, this simple, quick, and practical approach could be implemented across many other CNS and non-CNS conditions associated with pronounced placebo responses. The PCRS content can be easily translated for use in other languages and adjusted to fit the characteristics of unique indications and the trial’s methodology/design. The PCRS approach is also particularly amenable for tailored use in trials that incorporate electronic Clinical Outcome Assessment platforms. For example, the PCRS can be easily incorporated within a rater surveillance vendor’s tablet and can be verified as having been read to participants before administration of the primary efficacy scale. Audio recordings can also be reviewed to ensure that raters are properly administering and querying participants about the PCRS, while also ensuring the subjects are accurately summarizing the PCRS content. By enhancing signal detection, such procedures may help drug developers progress compounds to faster approval and reach patients who are suffering sooner.

## Funding and disclosure

The Placebo-Control Reminder Script (PCRS) is licensed to several pharmaceutical companies. Drs EAC and HHH are full-time Principal Investigators at Hassman Research Institute (HRI), which conducts clinical trials supported by the pharmaceutical industry. Dr LE receives support from APEX Innovative Services, which conducts research for most pharma, and is a performing site for NIDA, and through Follow the Molecule LLC receives consulting compensation from Biogen, Bioxcel, Neurocrine, Taisho, Atlas Investments, Athira, Intracellular, Cerevel, and Karuna. Dr VMG is a full-time Sub-Investigator at Collaborative Neuroscience Network (CNN), which conducts clinical trials supported by the pharmaceutical industry. Dr DPW is a full-time Principal Investigator at CNN, which conducts clinical trials supported by the pharmaceutical industry. Dr RSEK owns VeraSci which provides consulting and support services for many pharmaceutical companies. Dr KW has no disclosures. Dr WPH is a full-time employee of VeraSci.

## Supplementary information

Supplemental Material
