# Supplementary material for: Placebo response mitigation with a participant-focused psychoeducational procedure: a randomized, single-blind, all placebo study in major depressive and psychotic disorders
Source: Neuropsychopharmacology. 2020 Nov 26;46(4):844–50. doi: 10.1038/s41386-020-00911-5 (PMC8026614; doi:10.1038/s41386-020-00911-5)
Supplement: Supplementary file 1 — Supplemental Material [file 41386_2020_911_MOESM1_ESM.docx]

**Supplement**

**Mixed Model for Repeated Measures Analyses**

Change in BDI-II scores from Visit 1 to Visit 3 was analyzed using a mixed-effects model for repeated measures (MMRM) using all available data from randomized subjects (modified intent-to-treat sample; N = 137). The MMRM included visit, group (PCRS vs. NG), and the visit-by-group interaction as fixed effects; the stratification variables study center, sex, age (<40 vs. >/= 40) and diagnosis (SCZ vs. MDD) as fixed effects; visit as a repeated measure; baseline BDI-II as a covariate; and subject as a random effect, using an unstructured covariance matrix to model the covariance of within-patient scores. To examine whether the pattern of results was impacted by diagnosis, the three-way interaction of visit-by-group-by-diagnosis was added to the MMRM as a fixed effect.

Consistent with the RM-ANOVAs, the PCRS group showed a smaller decrease in BDI-II scores across visits than the NG. As shown in Supplemental Table 1 and Figure 1, the difference between groups on BDI-II changes from Visit 1 to Visit 3 was statistically significant (group-by-visit interaction: t[257] = 2.18, p = .03). Diagnostic status did not impact the overall pattern of BDI-II results (condition-by-visit-by-diagnosis interaction: t[258] = 1.00, p = .30).

**Supplemental Table 1**: Results from MMRM of BDI-II scores across visits

|  | Estimate | SE | df | t value | p-value | 95% CI |
| --- | --- | --- | --- | --- | --- | --- |
| Intercept | 6.10 | 2.05 | 147.85 | 2.97 | 0.00 | 2.05 to 10.15 |
| Visit | -0.66 | 0.07 | 256.10 | -9.07 | 0.00 | -.88 to -.52 |
| Group | 0.10 | 1.05 | 338.23 | 0.09 | 0.92 | -1.97 to 2.17 |
| Visit X Group | 0.22 | 0.10 | 256.91 | 2.18 | 0.03 | -.02 to 0.42 |
| BDI-II at Visit 1 | 0.77 | 0.06 | 127.75 | 13.46 | 0.00 | .65 to .88 |
| Sex | 0.30 | 0.76 | 128.24 | 0.39 | 0.70 | -1.20 to 1.80 |
| Site | 0.93 | 0.81 | 129.20 | 1.16 | 0.25 | -0.66 to 2.53 |
| Age | 0.60 | 0.84 | 128.18 | 0.71 | 0.48 | -1.07 to 2.26 |
| Diagnosis | 0.24 | 0.92 | 130.30 | 0.27 | 0.79 | -1.57 to 2.05 |

**Supplemental Figure 1**: BDI-II scores (least squares means) across visits
